# Dataset of protein changes induced by cold acclimation in red clover (*Trifolium pratense* L.) populations recurrently selected for improved freezing tolerance

**DOI:** 10.1016/j.dib.2016.06.003

**Published:** 2016-06-14

**Authors:** Marie Bipfubusa, Solen Rocher, Annick Bertrand, Yves Castonguay, Jenny Renaut

**Affiliations:** a Agriculture and Agri-Food Canada, Québec City, Canada; b Luxembourg Institute of Science and Technology, Belvaux, Luxembourg

**Keywords:** Red clover, Cold acclimation, Proteomic analysis, Recurrent selection, Freezing tolerance, Plant abiotic stress

## Abstract

The data provide an overview of proteomic changes in red clover (*Trifolium pratense* L.) in response to cold acclimation and recurrent selection for superior freezing tolerance. Proteins were extracted from crowns of two red clover cultivars grown under non-acclimated or cold-acclimated conditions, and plants obtained from the initial genetic background (TF0) and from populations obtained after three (TF3) and four cycles (TF4) of recurrent selection for superior freezing tolerance. Proteins were analyzed using a two-dimensional fluorescence difference gel electrophoresis (2D-DIGE) coupled to mass spectroscopy (MS and MS/MS). Differentially regulated proteins were subsequently identified using MALDI TOF/TOF analysis. The data are related to a recently published research article describing proteome composition changes associated with freezing tolerance in red clover, “A proteome analysis of freezing tolerance in red clover (*Trifolium pratense* L.)” (Bertrand et al., 2016 [1]). They are available in the ProteomeXchange Consortium database via the PRIDE partner repository under the dataset identifier PRIDE: PXD003689.

**Specifications Table**

**Table:** 

Subject area	**Biology.**
More specific subject area	**Plant proteomics.**
Type of data	Table, image.
How data was acquired	Proteins were separated using a two Dimensional fluorescence Difference Gel Electrophoresis (2D-DIGE) and analyzed by mass spectroscopy (MS and MS/MS). Matrix-assisted laser desorption/ionization (MALDI) peptide mass spectra were acquired using the AB Sciex 5800 time-of-flight (TOF) /TOF (AB Sciex, Redwood City, CA).
Data format	Analyzed, filtered.
Experimental factors	Comparative proteomic analysis of crowns of non-acclimated and cold-acclimated plants of two initial genetic background (Christie-TF0 and Endure-TF0) and populations obtained after three (TF3) or four cycles (TF4) of recurrent selection for superior freezing tolerance within each genetic background.
Experimental features	Proteins were separated using 2D-DIGE and analyzed using MS, and the differentially regulated proteins (regarding selection for superior freezing tolerance and cold acclimation status) were identified using MALDI TOF/TOF analysis.
Data source location	Quebec Research and Development Centre, Agriculture & Agri-Food Canada, Quebec, Canada. Luxembourg Institute of Science and Technology, Esch-sur-Alzette, Luxembourg.
Data accessibility	Data are within this article and available via the PRIDE partner repository on the ProteomeXchange Consortium database under the dataset identifier PRIDE: PXD003689.

**Value of the data**

• The first proteomic dataset of *Trifolium pratense* L.
• The data represent valuable tools to include in breeding programs aimed at improving freezing tolerance in Red clover.
• The data are useful to pursue further proteomic studies on the development of freezing tolerance in plants and to understand the cold acclimation process.
• Our proteomic data may be combined with genomic and transcriptomic data to provide better insights into the improved cold acclimation ability of recurrently selected red clovers for superior freezing tolerance.

## 1 Data

**Fig. 1:**
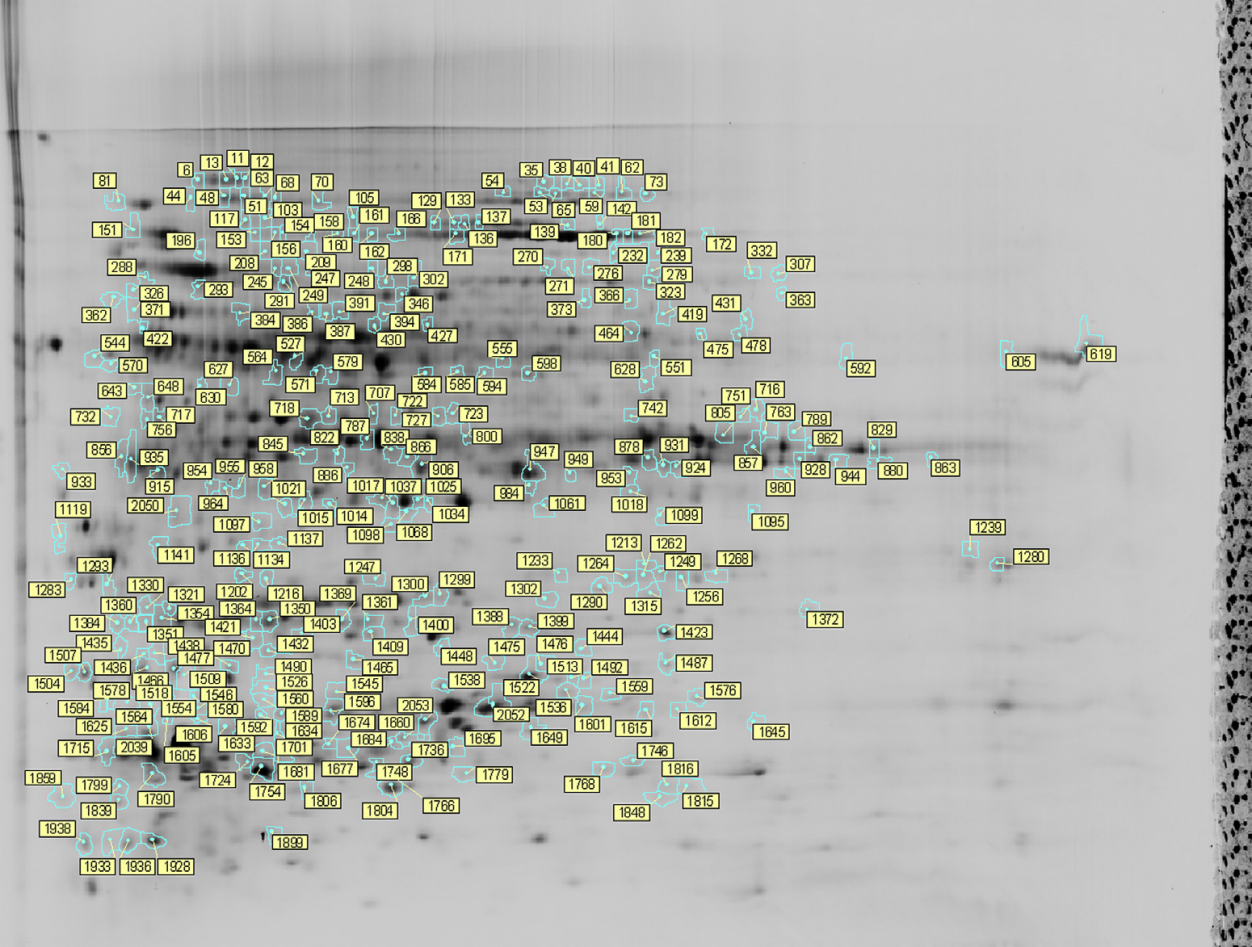
Master gel of two dimensional fluorescence difference gel electrophoresis (2D-DIGE) of total soluble proteins extracted from crowns of two red clover cultivars under cold acclimation and recurrent selection conditions. All spots with statistically significant abundance changes (ratios≥|1.5|, *p*<0.01) are labelled. Spot annotations refer to protein identifications provided in Supplemental Table 1. See Bertrand et al. [1] for data on protein abundance ratios and statistical analyses.

The data provide an overview of changes in crown proteome that are associated with the improvement of freezing tolerance in red clover (*Trifolium pratense* L.) through cold acclimation and recurrent selection for superior freezing tolerance [1]. Two dimensional fluorescence Difference Gel Electrophoresis (2D-DIGE) of total soluble proteins revealed about 1000 (992±54) validated spots per gel. The master gel is presented in Fig. 1. Protein showing abundance ratios ≥|1.5| with *P* values ≤0.01 for both ANOVA and *t*-test were subjected to similarity search against databases. Proteins successfully identified are listed in Supplemental Table 1 and their responses to cold acclimation and recurrent selection are reported.

## 2 Experimental design, materials and methods

### 2.1 Plant recurrent selection and cold acclimation

Plants were recurrently selected for superior freezing tolerance using a whole-plant screening approach that was described in [2]. Cold acclimation was performed following the procedure described in [3]. Briefly, plants of the two original genetic backgrounds (E-TF0 and C-TF0) and populations obtained after three (E-TF3 and C-TF3) and four cycles (E-TF4 and C-TF4) of recurrent selection were seeded in pots in September 2010, and allowed to establish in an environmentally controlled growth chamber under the following conditions: 16 h photoperiod; 600–800 μmol photons m^−2^ s^−1^ PPFD; 22 °C/17 °C (day/night). Plants were thinned to ten per pot after one week, and one half of pots were transferred to an unheated greenhouse for their acclimation to natural hardening conditions after 5 weeks of growth. Crowns were sampled before cold acclimation in October 2010 and after full cold acclimation in January 2011.

### 2.2 Protein extraction and quantification

Total soluble proteins were extracted from crowns using a trichloroacetic acid (TCA)/followed by a sodium dodecyl sulphate (SDS) and phenol extraction method as described in [4] with the following modifications. For each sample, 300 mg of ground lyophilized crowns was homogeneized in 1 mL of 20% (w/v) ice-cold TCA in acetone with 0.1% (w/v) dithiothreitol (DTT) and incubated for 60 min at −20 °C to allow protein precipitation. After centrifugation (10,000*g* for 5 min at 4 °C), pellets were washed with 1.5 mL ice-cold acetone and centrifuged again (10,000*g* for 5 min at 4 °C), dried under vacuum, and subsequently re-suspended in 0.6 mL UltraPure™ Buffer-Saturated Phenol (Invitrogen) and 0.6 mL SDS buffer [30% (w/v) sucrose, 2% (w/v) SDS, 0.1 M Tris–HCl, pH 8.0, 5% (v/v) 2-mercaptoethanol]. Samples were centrifuged (10,000*g* for 5 min at 20 °C), and 300 μL of the phenolic phase were mixed with 1.5 mL of ice-cold 0.1 M ammonium acetate in methanol and incubated for 30 min at −20 °C to allow protein precipitation. Pellets obtained after centrifugation (10,000*g* for 5 min at 4 °C) were washed twice with ice-cold 0.1 M ammonium acetate in methanol, and two more times with ice-cold acetone/water (80/20 (v/v)). Proteins were re-suspended in labelling buffer [7 M urea, 2 M thiourea, 30 mM Tris, 2% (w/v) 3-[(3-Chlolamidopropyl) dimethylammonio]-1-propanesulfonate (CHAPS)]. Samples were shaken on an Eppendorf Thermomixer (700 rpm for 60 min at 20 °C) and centrifuged (5 min at 14,000*g*) to remove insoluble material. The pH of the supernatant was adjusted to 8.5 using 1 M NaOH and protein concentration was determined with a Bradford protein assay using bovine serum albumin (2 mg mL^−1^) as standard [5].

### 2.3 Two-dimensional difference gel electrophoresis (2D-DIGE)

Protein separation was performed using the 2D-DIGE approach described in [6] with some modifications. For each sample, 50 μg of proteins were labelled with Cy3 and Cy5 dyes according to the manufacturer׳s instructions (CyDyes LUMIPROBE LLC 25 nmol (Interchim®), mixed with a Cy2-labelled internal standard, and the volume adjusted to 150 μl with a rehydration buffer [7 M urea, 2 M thiourea, 0.5% (w/v) CHAPS]. Proteins were first separated on IPGs of a non-linear 3–10 pH range using an Ettan IPGphor III system (GE Healthcare). IPG strips were equilibrated for 15 min in an equilibration buffer (Serva Electrophoresis GmbH, Heidelberg) complemented with 6 M Urea and 1% w/v DTT and the second dimensional electrophoresis was carried out on a 12.5% SDS-PAGE using an HPE™ Flatbed Tower System (Serva Electrophoresis GmbH). The gels were matched and scanned using a Typhoon FLA 9500 scanner (GE Healthcare, Diegem, Belgium), and quantified using the DeCyder software (v7.0, GE Healthcare, Diegem, Belgium).

### 2.4 Protein digestion and MALDI-TOF_TOF analyses

Differentially regulated spots were excised with an Ettan Spot Picker robot (GE Healthcare), tryptically digested and spotted on the MALDI target using the Tecan freedom EVO200 (Tecan, Männedorf, CH). MALDI peptide mass spectra were acquired using the AB Sciex 5800 time-of-flight (TOF)/TOF (AB Sciex, Redwood City, CA). For subsequent MS/MS analysis, ten most intense peaks were selected and fragmented. Protein identification of MS and MS/MS spectra was initially searched against the NCBI non-redundant protein sequence database (http://www.ncbi.nlm.nih.gov) downloaded on June 6th, 2014 reduced to the green plants Taxonomy using an in-house MASCOT server (Matrix Science, www.matrixscience.com, London, U.K.). Unmatched peptides with low significance score were further subjected to similarity searches against an EST *Fabaceae* database downloaded from the NCBI server on December 17th, 2013. The Mascot database search allowed up to two missed cleavages and a mass tolerance of ±100 ppm for peptides and ±0.5 Da for fragments. Carbamidomethylation of cysteine was set as fixed modification while oxidation of methionine, simple and double oxidation of tryptophan and oxidation of tryptophan to kynurenine were selected as variable modifications. Proteins were considered as successfully identified when at least two peptides had a score above the MASCOT-calculated 0.05 threshold score of 40.

### 2.5 Statistical analyses

The average values of four biological replicates (1 rep=1 pot, 10 plants per pot) were used to compare the protein abundances between different treatments. For each protein spot, changes in abundance in response to cold acclimation and recurrent selection were tested by one-way and two-way ANOVA combined with a Student׳s *t*-test. Spots with *P*-values ≤0.01 in ANOVA and *t*-test and fold changes ratios ≥|1.5| were considered to be differentially regulated.

## Appendix A Supplementary material

Supplementary material Mass=mass of the intact protein according to the databank; score=score of the protein according to MASCOT, the probability based score according to the number of matches and the database size; Expect=expected value that this protein can appear by chance; matches=number of peptide masses that are matching with the in silico digest in the database for this particular protein; ppm=difference between the calculated mass of the uncharged precursor and the theoretical mass; Mr(expt)=theoretical mass: mass calculated based on the matched peptide sequence; Mr(calc)=calculated mass: mass of the selected precursor minus one proton, corresponds to the precursor with no charge; Start-End: part of the protein sequence covered by the matched peptide; Ions=score of the peptide; miss= number of missed cleavages.
