# Supplementary material for: Dataset of protein changes induced by cold acclimation in red clover (Trifolium pratense L.) populations recurrently selected for improved freezing tolerance
Source: Data Brief. 2016 Jun 14;8:570–4. doi: 10.1016/j.dib.2016.06.003 (PMC4927546; doi:10.1016/j.dib.2016.06.003)
Supplement: Supplementary file 1 — Supplementary material [file mmc1.docx]

**DIB-D-16-00341 – Conflict of interest statement**

The authors declare that they have no conflict of interest
